# Perihepatitis in systemic lupus erythematosus

**DOI:** 10.11604/pamj.2015.20.280.5534

**Published:** 2015-03-23

**Authors:** Janet Laabidi, Faida Ajili

**Affiliations:** 1 Department of Internal Medecine, Military Hospital of Tunis, 1008 Montfleury, Tunisie

**Keywords:** Lupus erythematosus systemic, pelvic disease inflammatory, perihepatitis

## Image in medicine

We report the case of a 42-year old male patient was admitted to our hospital because of a two weeks history of severe dry cough and right upper quadrant pain. He had been diagnosed as LES with diffuse proliferative lupus nephritis (class IV) in 2006 treated intially by intravenous steroid pulse therapy combined with an immunosuppressant and benefited in 2007 of Hemodialysis. In July 2013, he suffered of constant abdominal pain located at the right upper quadrant with dry cough. C-reactive protein (CRP) was 17 mg/L (normal < 8 mg/L) on admission with positive antinuclear antibodies title at 1/1280. The chest-abdominal computed tomography scan showed a pleural effusion a regular hepatomegaly with a rim of enhancement involving the right lobe of the liver. No ascites was present and there was no ancillary evidence of cholecystitis. The findings were thought to be most consistent with a perihepatitis. Pleural fluid examination showed an exudative pleural effusion with a negative culture. The diagnosis of perihepatitis associated with SLE complicated by a right pleural effusion was taken. The patient condition was treated with two antibiotics (vibramycin and amikacyn) and oral prednisolone. On review 4 weeks later his pain, cough and the right pleural disappeared. Perihepatitis is a rare disease, normally associated with pelvic inflammatory disease. It has rarely been reported in association with systemic lupus erythematosus (SLE) particularly in patients with lupus hemodialysis nephritis.

**Figure 1:**
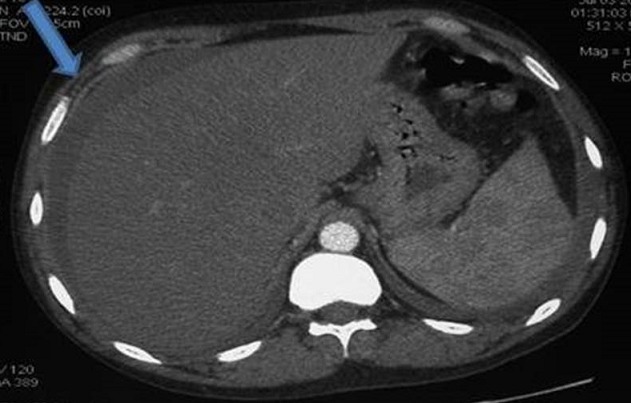
Irregularly enhancing rim along the anterolateral aspect of the right lobe of the liver (arrow), consistent with perihepatitis

